# Pharmacoeconomics in Africa: needs, prospect and challenges

**DOI:** 10.1186/s40545-021-00328-5

**Published:** 2021-05-31

**Authors:** Temitope Ben-Ajepe, Ifechukwu Benedict Nwogu, Damilola Quazeem Olaoye, Abdulhafeez Ayodele Mustapha, Theogene Uwizeyimana, Yusuff Adebayo Adebisi

**Affiliations:** 1 Dora Akunyili College of Pharmacy, Igbinedion University, Okada, Edo Nigeria; 2 Advantage Health Africa, Lagos, Nigeria; 3 Faculty of Pharmacy, University of Ibadan, Ibadan, Nigeria; 4 Faculty of Pharmacy, Obafemi Awolowo University, Ile-Ife, Nigeria; 5 Department of Public Health, Mount Kenya University Rwanda, Kigali, Rwanda; 6 Faculty of Pharmacy, University of Ibadan, Ibadan, Nigeria; 7 Medical Research Center, Kateb University, Kabul, Afghanistan

**Keywords:** Pharmacoeconomics, Needs, Prospect, Challenges, Africa

## Abstract

Africa as a continent has experienced a continuous increase in the cost of healthcare as its demands increase. With many of these African countries living below the poverty threshold, Africans continue to die from preventable and curable diseases. Population increases have led to an increase in demands for healthcare, which unfortunately have been met with inequitable distribution of drugs. Hence, the outcomes from healthcare interventions are frequently not maximized. These problems notably call for some economic principles and policies to guide medication selection, procurement, or donation for population prioritization or health insurance. Pharmacoeconomics drives efficient use of scarce or limited resources to maximize healthcare benefits and reduce costs. It also brings to play tools that rate therapy choice based on the quality of life added to the patient after a choice of intervention was made over an alternative. In this paper, we commented on the needs, prospect, and challenges of pharmacoeconomics in Africa.

## Introduction

The cost of healthcare keeps increasing as there is a continuous increase in the demand for its services. These increases can be well attributed to the introduction of sophisticated healthcare technologies, drug costs, increasing standard of living, and increasing demand for better individual tailored, quality healthcare services [1]. The shortage in supply of drugs and lack of regular access to essential medicines in low- and middle-income countries inevitably calls for some economic principles [2, 3]. Interestingly, many African countries lack policies guiding medication selection, procurement, or donation for population prioritization or health insurance [4]. In countries where these policies are available, there is a lack of consistency in their application during decision-making processes [5–7]. Countries worldwide have applied pharmacoeconomic principles in identifying, measuring, and comparing the cost and benefits of pharmacotherapies. Pharmacoeconomics aims for efficient use of limited resources for the maximization of healthcare benefits and reduced costs. These aims and applications solve many problems affecting access, supply, and equitable distribution of medication in African countries.

Pharmacoeconomics brings a cost–effectiveness approach to drug therapy. It gives rise to a better distribution of healthcare resources and their overall utilization.

Pharmacoeconomic studies often employ four techniques, namely cost-minimization analysis—which focuses on costs of treatment and ignores benefits. Cost–effectiveness analysis compares costs of therapy in monetary units to the effectiveness of such therapy in natural clinical units. Cost–benefit analysis compares costs to the recipient’s accrued advantages. The last technique is the cost–utility analysis that compares the cost to unit satisfaction achieved from therapy choice [8]. The typical outcome metric in cost–effectiveness analysis when making informed therapy choice and resource distribution decisions are Quality-Adjusted Life Years (QALY). It rates therapy choice based on the quality of life (QoL) added to the patient after a choice of intervention was made over an alternative. Hence, the costs and outcomes of a therapy can be juxtaposed. These, if brought into the African context, will solve many problems on waste and uninformed utilization and distribution of healthcare resources. This commentary discusses the needs, prospects, and challenges of pharmacoeconomics in Africa.

## Needs of pharmacoeconomics in Africa

According to the World Bank, Africa consists of low, lower middle, upper-middle, and high-income countries. A sizable number of these African countries live below the poverty line. Due to Africa's poverty level, people continue to die from preventable and curable diseases that can be controlled if there was easy access to medications, vaccines, and other medical services. A significant percentage of drugs used in Africa are imported and not locally produced, making it relatively unaffordable for the larger population [9]. This population relies on public health services, which are continuously running low on drugs and services in comparison to the high demand for it. Africa has an overwhelming amount of contagious and non-contagious diseases, however is also constrained on limited resources to address them [10, 11]. The pharmacoeconomics concept's primary driver is the ability to provide full health services within a "limited budget" [12]. Even though 30–60% of healthcare budgets in low-income countries are allocated to drugs, the scarcity is still attributed to inadequate funds allocation to pharmaceuticals. This circumstance suggests the high need for pharmacoeconomic concepts in Africa [12]. The diseases require more effective medications for treatment in the past couple of decades, raising the total cost of healthcare [13]; hence, the significance for pharmacoeconomics cannot be overemphasized.

Medical resolution in developed societies is usually significantly influenced by "health economics"; this influence also affects doctor's medical practice and formulation choices by pharmacists. The cost-effective allocation of health inputs could potentially increase service delivery and improve access within existing budgets [14]. It has been shown that many developing countries, including Africa, lack health economics considerations, even when in the most need of it [12]. There is nonexistence of policies that push for economic assessment in medicine choices be it for public subsidizing, setting up aid, or healthcare coverage [12]. Significant rules for detailing pharmacoeconomics examinations are not accessible in most countries. In places where they exist, they are not logically considered in "decision-making". Experience shows that numerous pharmacoeconomic contemplates were directed long after authorities had already made the choices. This process is done once in a while essentially to either legitimize or preclude those choices selected or more frequently for scholarly purposes [12]. For instance, a recent study in four centers in sub-Saharan African countries revealed that childhood cancer treatment units represent cost-effective interventions, and this is very much relevant in developing national childhood cancer strategies across Africa [15]. A study in Zambia has also shown how pharmacoeconomics can be used in understanding the cost–effectiveness of cholera vaccination program [16]. All these reiterate the much-needed importance of pharmacoeconomics in Africa, a continent that is facing double burden of communicable and non-communicable diseases, with limited healthcare resources.

## Prospects of pharmacoeconomics in Africa

Given that many African countries are handicapped by very scarce resources, governments and policymakers are likely to explore effective means to maximize health and treatment outcomes with the limited funds available. As such, the application of pharmacoeconomic methods might prove to be a useful tool in guiding health expenditures and informing health policy decisions [12]. However, it is widely agreed that, in selecting medicines for national drug formulary in developing countries, pharmacoeconomic principles should not be blindly applied, but considered alongside other policy and contextual issues such as the structure and performance of the health system and the country’s pharmaceutical landscape.

In what stirred up a controversial scientific debate, Babar and Scahill proposed a model that questions the usefulness and applicability of pharmacoeconomics in formulary development, arguing that reference pricing and generic substitution may be better first-line tools compared to conventional cost–effectiveness and cost–utility techniques [17]. Proponents have hypothesized that this approach is likely to yield a higher return on investment compared to pharmacoeconomic methods and should therefore be given priority [18]. However, critics maintain that pharmacoeconomics has multi-level and complementary functions in guiding health policy decisions [17], and should be given equal consideration, rather than relegated. Our position is that pharmacoeconomics holds solid relevance in choosing drug therapies that offer better value for money, particularly in the context of assessing new, more expensive, yet more effective drug therapies. Moreover, we assert that pharmacoeconomics houses enormous potential in Africa—characterized by weak health systems and heightened healthcare needs in ensuring judicious use of scarce resources. Interestingly, it could also provide a means of reimbursement for health insurance companies, which are becoming increasingly common in many developing countries [12]. However, it is important to emphasize that, pharmacoeconomics should not be considered in isolation but applied complementarily and together with other cost-containment strategies, such as drug pricing policies and the use of generic substitutes, as part of a robust health decision-making strategy [12, 19].

## Challenges of pharmacoeconomics in Africa

Available data reveal a major dearth in pharmacoeconomic studies on the continent. Countries from the east and western parts of Africa document a very limited amount of this type of study that could be useful as evidence to inform and support decision-making at higher levels [20]. Furthermore, studies available were either too narrow in scope to be considered for comparing costs and consequences of drug therapy to healthcare systems and the society overall [19]. Important information comparing the clinical effectiveness of pharmaceuticals to generic alternatives which serve as the foundation of any useful pharmacoeconomic study is glaringly absent [20]. In addition, a study conducted among senior pharmacists, consultant physicians, and clinical pharmacologists in 12 leading tertiary healthcare facilities in Nigeria revealed that lack of expertise is a challenge to perform any form of economic evaluation of medicines [21]. This is similar to findings from South Africa, where lack of expertise is a challenge [22].

Trends for the available pharmacoeconomic studies depict that as opposed to them being actively involved throughout the decision-making process, were featured when decisions had already been made either in an effort to justify or disqualify said decision(s) [23]. As a result, the impact of such studies was purely academic and had no real influence on policies or implementation whatsoever. Difficulties inclusive of continued lack of access to essential, generic medication are rampant in many parts of the continent which is responsible for a large percentage of the world’s poor and underserved citizens and encumbered with a disproportionate share of the disease burden [24], but account for 1% of total global expenditures [25]. This is upheld by the lack of evidence-based frameworks for improved financing strategies [26].

## Conclusion

While pharmacoeconomics is gradually gaining traction in Africa, more actions are required in the areas of financing, advocacy, education and training, and policy implementation. Considering that pharmacoeconomic analyses are not inexpensive, there is a need for a better commitment from African governments and institutions in funding more, high-quality economic evaluations of drugs and pharmaceutical services. In addition, deliberate steps should be taken to incorporate pharmacoeconomics into the curriculum of medical and pharmacy schools, including hosting short courses and seminars. Finally, policies should be implemented to promote increased use of cost–effectiveness data to drive evidence-based health decisions in Africa.

## Data Availability

Not applicable.

## Abbreviations

QALY: Quality-Adjusted Life Years
QoL: Quality of Life
